# Clinical Spectrum and Treatment Outcomes of Rare Bleeding Disorders in Female Patients: A Two-Center Experience in North Pakistan

**DOI:** 10.7759/cureus.77533

**Published:** 2025-01-16

**Authors:** Muhammad Usman, Nighat Shahbaz, Mehreen Ali Khan, Hira Tariq, Rafia Mahmood, Saad Jamshed, Raheel Iftikhar, Mehwish Gilani, Maryum Khan, Tahira Zafar

**Affiliations:** 1 Clinical Hematology, Armed Forces Bone Marrow Transplant Centre Rawalpindi, Rawalpindi, PAK; 2 Hematology, Armed Forces Bone Marrow Transplant Centre Rawalpindi, Rawalpindi, PAK; 3 Epidemiology, Armed Forces Bone Marrow Transplant Centre Rawalpindi, Rawalpindi, PAK; 4 Hematology, Armed Forces Institute of Pathology, Rawalpindi, PAK; 5 Hematology and Oncology, Rochester Regional Health, Rochester, USA; 6 Hematology and Oncology, Armed Forces Bone Marrow Transplant Centre Rawalpindi, Rawalpindi, PAK; 7 Chemical Pathology, Armed Forces Bone Marrow Transplant Centre Rawalpindi, Rawalpindi, PAK; 8 Hematology, Hemophilia Treatment Center, Rawalpindi, PAK

**Keywords:** clinical spectrum, factor deficiency, north pakistan, rare bleeding disorders, women with rbds

## Abstract

Introduction

Rare bleeding disorders (RBDs) result from genetic mutations in clotting factors. These RBDs vary in prevalence and are often underdiagnosed due to mild symptoms. Treatment is challenging due to limited clinical data and primarily involves substituting deficient factors and using adjuvant therapies. Women with RBDs face unique risks, including gynecologic bleeding, hemorrhagic ovarian cysts, and complications during pregnancy. These issues can significantly impact their quality of life and employment. This study was conducted to characterize the patterns of bleeding disorders, clinical manifestations, and treatment outcomes in female patients.

Methods

In this cross-sectional study, we included patients from the Hemophilia Treatment Center (HTC) and Armed Forces Bone Marrow Transplant Centre (AFBMTC) Rawalpindi between 2011 and 2023, using a convenience sampling technique. Data were extracted from patient files, including medical history, factor activity levels, symptoms, treatments, and medications. Eligible participants had congenital coagulation factor deficiencies, while those with platelet function or acquired coagulation disorders were excluded.

Results

In our study of 50 patients with RBD, the median age at bleeding presentation was two years; 72% of cases were born of consanguineous marriages, and 57% had a positive family history of bleeding disorders. Factor V deficiency was the most prevalent (28%), and major bleeding episodes occurred in 52% of cases. The predominant clinical presentations included menorrhagia (74%) and epistaxis (58%). Treatment primarily involved antifibrinolytics (98%) and FFP transfusions (96%), with significant associations identified among various risk factors related to bleeding. All patients were counseled regarding local measures for bleeding control. There was a moderate correlation found between factors V and VII with the International Society on Thrombosis and Haemostasis Bleeding Assessment Tool (ISTH BAT) score. There is a weak correlation between factors X and XI with the ISTH BAT score. There was no correlation found in factor I, factor XI, factor XII, and combined factors V+VIII deficiency.

Conclusion

Women with RBDs face a spectrum of bleeding challenges, significantly impacting their quality of life and reproductive health. Early diagnosis and personalized treatment strategies are paramount in mitigating bleeding risks and enhancing patient outcomes.

## Introduction

Rare bleeding disorders (RBDs) are a group of diseases that are caused by a genetic mutation in one or more clotting factors, such as factor VII (FVII), factor X (FX), factor XI (FXI), factor XIII (FXIII), factor I (FI), factor II (FII), factor V (FV), combined FV and factor VIII (FVIII), and vitamin K-dependent clotting factors. These diseases are genetic in an autosomal recessive pattern [1,2]. The frequency of such disorders varies globally, ranging from 1:500,000 for FVII insufficiency (FVIID) to 1:2 million for FII (FIID) and FXIII deficiency (FXIIID). According to data from the World Federation of Hemophilia and the European Network of Rare Bleeding Disorders, the most common deficiencies among the entire affected population were FVII (39%) and FXI (26%), fibrinogen, FV, and FX (8-9%), FXIII (6%), and combined FV and FVIII (3%); FII deficiency (prevalence, 1%) was the rarest disorder [3]. RBD treatment is challenging due to the lack of clinical management information regarding specific bleeding episodes. The primary stay of treatment involves substituting the coagulation factor that is deficient and, where necessary, using adjuvant therapy (antifibrinolytics, estrogen/progestogen) [4-6].

As a developing country with limited healthcare resources, Pakistan struggles to provide comprehensive diagnostic services for bleeding disorders. Women with RBDs frequently experience not only classic bleeding symptoms but also gynecological complications necessitating specialized clinical management. Such complications may include hemorrhagic ovarian cysts, endometriosis, endometrial hyperplasia, uterine polyps, fibroids, and menorrhagia. Additionally, certain bleeding disorders are associated with increased risks of miscarriage, antenatal bleeding, and postpartum hemorrhage (PPH). These challenges, particularly during pregnancy and childbirth, can adversely affect the employment prospects and overall quality of life of affected individuals [7,8].

It has been recognized that there is a critical need to better understand the clinical spectrum of these disorders in female patients to improve early diagnosis, guide appropriate management, and optimize treatment outcomes in our population. This study was conducted to characterize the patterns of bleeding disorders in female patients and to document the clinical manifestations in this cohort.

## Materials and methods

The study utilized a retrospective, cross-sectional design, focusing on patients who were registered at the Hemophilia Treatment Center (HTC) in Rawalpindi and those treated at the Armed Forces Bone Marrow Transplant Centre (AFBMTC) between 2011 and 2023. This design allowed for a comprehensive examination of historical data from a range of patient records within a defined time frame, offering insights into the clinical characteristics and treatment outcomes of patients with bleeding disorders. Data were meticulously retrieved from the patient records, ensuring that all relevant information, such as medical history, baseline factor activity levels, clinical manifestations, treatment protocols, and concurrent medications, was captured comprehensively. This thorough approach ensured that the study reflected the real-world clinical environment and provided a robust dataset for analysis.

The study adhered strictly to the ethical principles outlined in the World Medical Association's Declaration of Helsinki for studies involving human subjects. This ensured that the research was conducted with the highest ethical standards, protecting patient confidentiality and promoting informed consent while ensuring that patient welfare remained paramount throughout the study process. Institutional Review Board (IRB) approval was sought from the relevant committees at both the HTC and AFBMTC, ensuring ethical compliance.

To assess the clinical outcomes, patients’ bleeding events were categorized into major and minor types based on standardized definitions. Major bleeding events were defined as those involving significant clinical impact, including the following: intracranial, intraocular, or retroperitoneal bleeding, overt blood loss associated with a hemoglobin decrease greater than 3 g/dL, any hemoglobin decrease greater than 4 g/dL. There is a need for a transfusion of two units of blood products or more. In contrast, minor bleeding was defined as overt bleeding that did not meet the criteria for major bleeding, such as minor skin bleeds or mucosal bleeding that did not result in significant blood loss or require transfusion [7].

Inclusion and exclusion criteria

This study recruited participants with congenital coagulation factor deficiencies based on well-defined criteria. Eligibility was determined by coagulation factor activity levels below the normal range, with diagnoses supported by indicators such as documented bleeding tendencies, family history evaluations, or abnormal laboratory findings during routine screening. To maintain the study's focus on congenital deficiencies, exclusion criteria were rigorously applied, excluding individuals with platelet function disorders or acquired coagulation abnormalities.

Statistical analysis

In the descriptive analysis, percentage and frequency were calculated for qualitative variables and median and interquartile range (IQR) for all the quantitative variables. Correlation analysis was done to correlate the factors deficiency with the International Society on Thrombosis and Haemostasis Bleeding Assessment Tool (ISTH-BA) scores. The strength of the correlations was described by using the following classification: no correlation (0-0.29), weak correlation (0.30-0.59), moderate correlation (0.50-0.69), strong correlation (0.70-0.89), and very strong correlation (0.90-1.0). p < 0.05 was considered statistically significant.

## Results

The study group comprised 50 female patients diagnosed with RBDs. The median age at first bleeding presentation was two years (IQR: 1-8 years), while the median age at diagnosis was three years (IQR: 2-10 years). When stratified by age groups, nine patients (19%) were aged 0-10 years, 22 (47%) were aged 11-20 years, and 16 (34%) were aged 21-30 years. A significant proportion of the cohort, 36 patients (72%), were born to consanguineous marriages, and 26 (57%) reported a positive family history of bleeding disorders in a first-degree relative.

**Figure 1:**
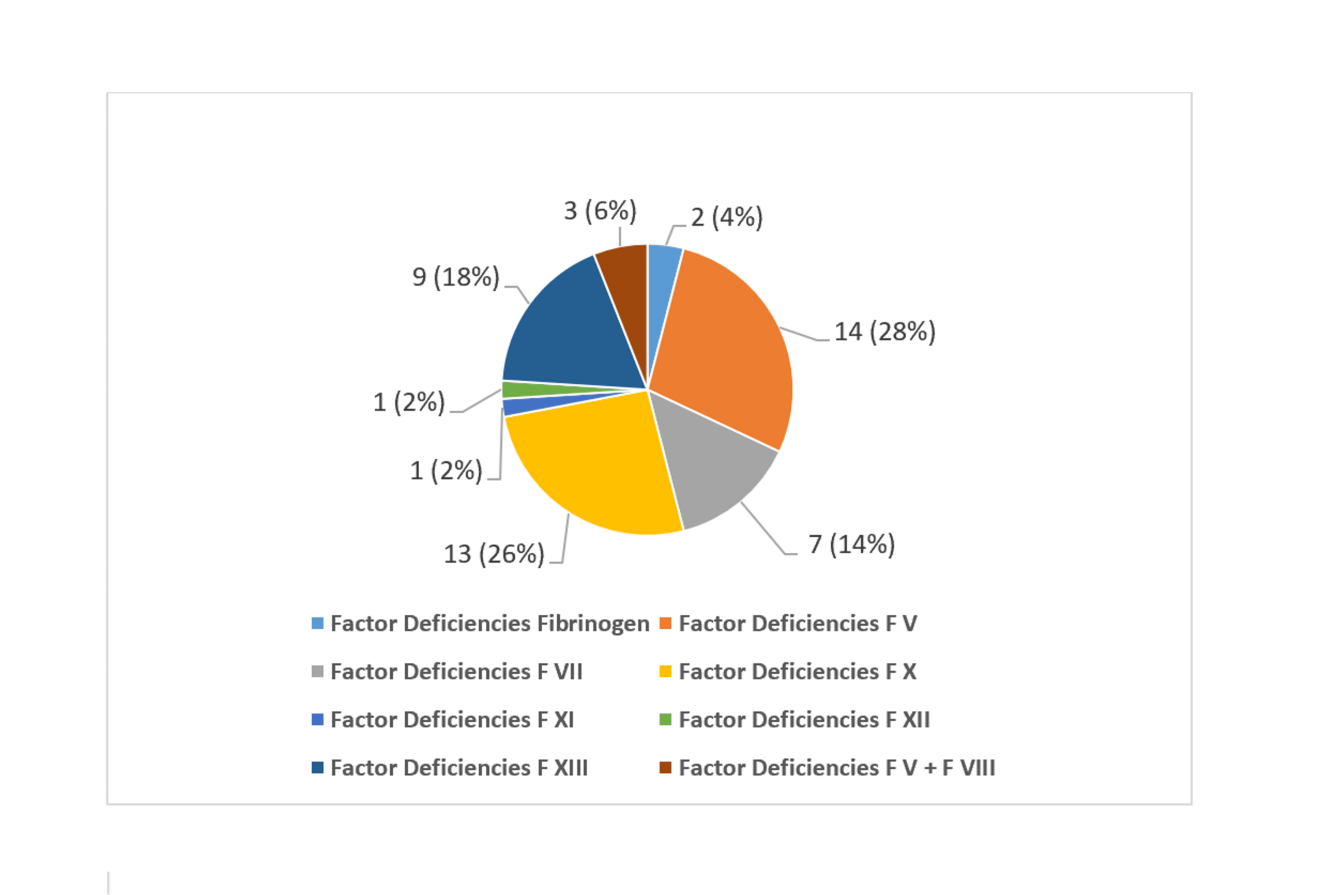
Distribution of rare bleeding disorders

Notably, 26 patients (52%) experienced at least one episode of major bleeding, as defined by the ISTH criteria. Among the cohort, FV deficiency emerged as the most prevalent condition, identified in 14 patients (28%) (Figure 1).

These findings underscore the early onset, familial clustering, and diagnostic challenges associated with RBDs, particularly in populations with high rates of consanguinity.

The most common clinical presentation was menorrhagia in 37 (74%) patients, epistaxis in 28 (58%), and skin bruising in 26 (52%) patients, followed by gum bleeding in 24 (48%) patients. By using chi-square, a significant association was found between various risk factors like family history, umbilical stump bleeding, bleeding, and epistaxis with the factor levels (Table 1).

**Table 1:** Factors deficiency with signs and symptoms FI, factor I; FVII, factor VII; FVIII, factor VIII; FV, factor V; FX, factor X; FXI, factor XI; FXII, factor XII; FXIII, factor XIII

	FI	FV	FVII	FX	FXI	FXII	FXIII	FV+FVIII
	n(%)	n(%)	n(%)	n(%)	n(%)	n(%)	n(%)	n(%)
Umbilical stump bleed	1 (50)	0 (0)	1 (14)	2 (15)	0 (0)	0 (0)	8 (89)	2 (67)
Epistaxis	1 (50)	10 (7)	1 (14)	6 (46)	0 (0)	0 (0)	7 (78)	3 (100)
Skin bruising	2 (100)	8 (57)	1 (14)	7 (54)	0 (0)	0 (0)	6 (67)	2 (68)
CNS bleeding	1 (50)	3 (21)	2 (29)	1 (8)	0 (0)	0 (0)	3 (33)	1 (33)
Gum bleed	1 (50)	8 (57)	1 (14)	7 (54)	0 (0)	0 (0)	5 (56)	2 (68)
Menorrhagia	2 (100)	11(79)	5 (71)	9 (70)	0 (0)	0 (0)	7 (78)	2 (68)
Musculoskeletal bleed	1 (50)	3 (21)	2 (29)	1 (8)	0 (0)	0 (0)	6 (67)	0 (0)
Hemarthrosis	1 (50)	1 (7)	1 (14)	2 (15)	0 (0)	0 (0)	1 (11)	0 (0)
Hematuria	0 (0)	0 (0)	0 (0)	1 (8)	0 (0)	0 (0)	0 (0)	0 (0)
Postpartum	0 (0)	0 (0)	0 (0)	1 (8)	0 (0)	0 (0)	2 (22)	0 (0)
Post-dental extraction	0 (0)	1 (7)	0 (0)	0 (0)	0 (0)	0 (0)	1 (11)	0 (0)
Pregnancy	0 (0)	1 (7)	0 (0)	2 (15)	0 (0)	0 (0)	1 (11)	0 (0)
GI bleed	0 (0)	4 (29)	0 (0)	1 (8)	0 (0)	0 (0)	6 (67)	0 (0)
Post-traumatic bleed	0 (0)	1 (7)	0 (0)	1 (8)	0 (0)	0 (0)	1 (11)	0 (0)
Post-surgical bleed	0 (0)	3 (21)	1 (14)	4 (31)	0 (0)	0 (0)	1 (11)	0 (0)
Delayed wound healing	0 (0)	1 (7)	0 (0)	2 (15)	0 (0)	0 (0)	3 (33)	0 (0)

**Figure 2:**
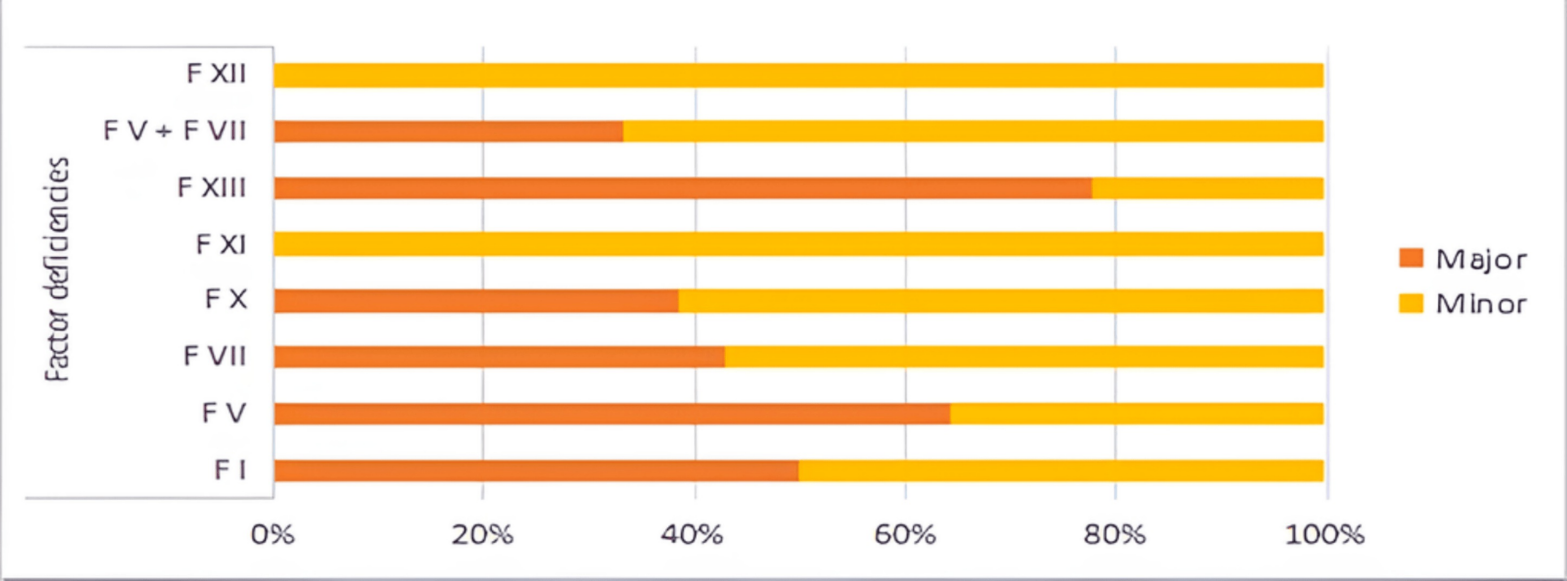
Bleeding severity of patients with factor deficiencies

Seventy-eight percent of patients had major bleeding in FXIII deficiency, 62% of patients with FV deficiency, while there was no bleeding in FXII deficiency (Figure 2).

**Figure 3:**
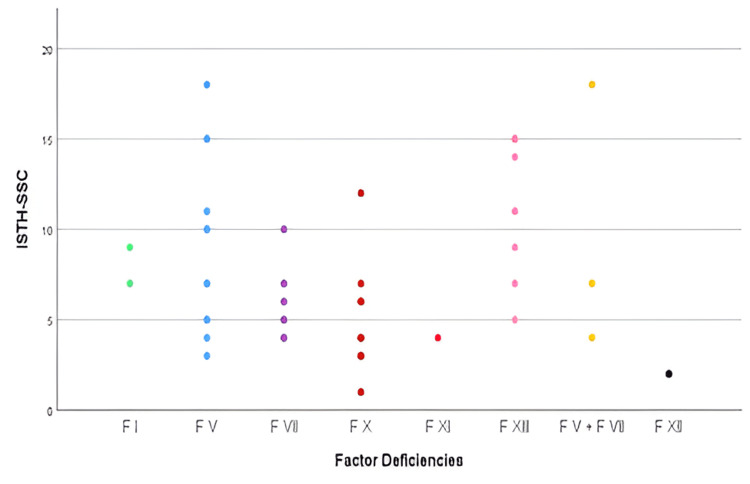
International Society on Thrombosis and Haemostasis Bleeding Assessment Tool (ISTH BAT) score with factor deficiencies

There were large differences in the BAT scores for each RBD. The highest median scores were seen in patients with FV and combined FV+FVIII deficiency, followed by FXIII (Figure 3).

**Figure 4:**
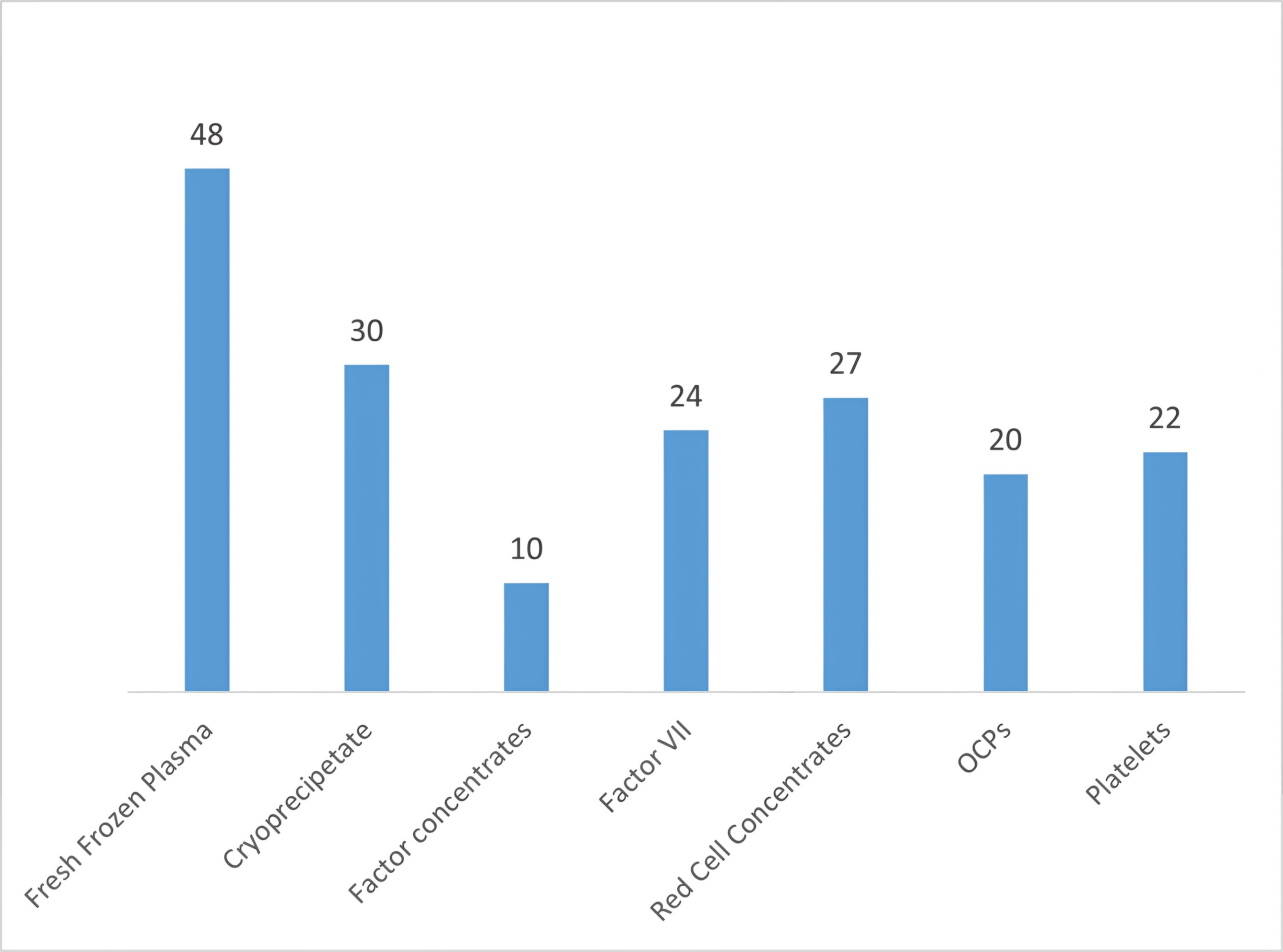
Treatment description

Antifibrinolytics were the mainstay treatment in 49 (98%) patients. Forty-eight (96%) had received FFP transfusions. Thirty (60%) patients received cryoprecipitate. Recombinant FVII (rFVIIa) was administered to 24 (48%) patients; of these, three females required it during delivery or cesarean section, and one received it for the management of PPH. Twenty-two (44%) patients received platelets, while 20 (40%) patients received oral contraceptive pills. All patients were counseled regarding local measures for bleeding control (Figure 4).

**Figure 5:**
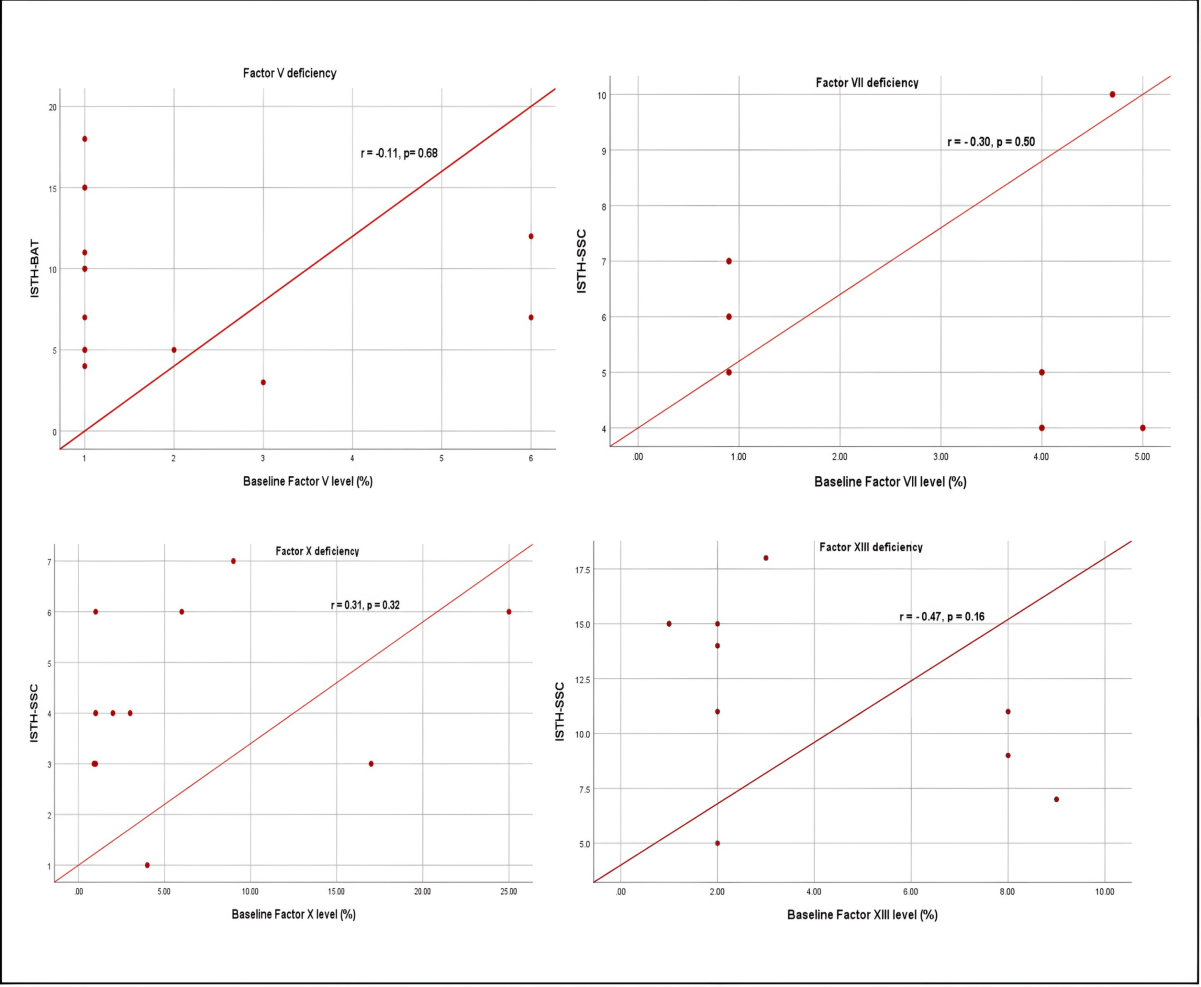
Correlation between baseline factor activity levels and International Society on Thrombosis and Haemostasis Bleeding Assessment Tool (ISTH BAT) score

The correlation analysis between coagulation factor levels and the ISTH-BAT score revealed varying relationships across different factor deficiencies (Figure 5). FV and FVII demonstrated a moderate correlation with the ISTH-BAT score, suggesting that their levels may be more predictive of bleeding severity. In contrast, FX and FXIII showed only a weak correlation, indicating that while there is some association, other elements likely play significant roles in determining bleeding risk for these deficiencies. Notably, no correlation was found between the ISTH-BAT score and deficiencies in fibrinogen (FI), FXI, FXII, or combined FV+FVIII. This lack of correlation implies that, for these factors, bleeding severity cannot be reliably predicted by factor levels alone. These findings highlight the complex nature of RBDs and emphasize the need for comprehensive clinical assessment. The variability in correlations across different factor deficiencies underscores the importance of individualized patient evaluation and management strategies.

## Discussion

RBDs are a heterogeneous group of uncommon conditions arising from deficiencies in specific coagulation factors, including FI (fibrinogen), FII (prothrombin), FV, FVII, FX, FXI, and FXIII [9,10]. Although individually rare, these disorders collectively represent a significant clinical challenge due to their varied presentations, diagnostic complexities, and management difficulties [11]. The diagnosis often requires advanced laboratory techniques, while treatment options may be limited, particularly in resource-constrained settings [12].

The impact of RBDs is particularly profound in females, as their reproductive health is intricately linked with bleeding risks. Menstrual cycles, pregnancy, childbirth, and gynecologic surgeries can exacerbate bleeding tendencies, leading to increased morbidity and potential complications. This dual interaction between hematologic abnormalities and gynecologic health amplifies the challenges in providing comprehensive care. Consequently, the management of RBDs in females necessitates a multidisciplinary approach that integrates expertise in hematology, gynecology, and reproductive medicine to ensure effective diagnosis, tailored treatment, and optimal quality of life [8,9].

In our cohort, FV deficiency was a common RBD, affecting 28% of patients, followed by FX deficiency at 26%. This contrasts with a study of Iran from the Fars Hemophilia Center, which reported FVII deficiency as the prevalent RBD, with FX deficiency being the second most common [13]. A retrospective study of 156 patients in Turkey also identified FVII and FV deficiencies as the most frequent RBDs [14]. The autosomal recessive inheritance pattern of many factor deficiencies makes RBDs more pronounced in regions with elevated consanguinity [15]. In our cohort, 72% of patients had consanguineous parentage, significantly higher than the 47.2% consanguinity rate observed in the Turkish RBD population [16].

Females with RBDs face unique challenges, especially in relation to menarche, menstrual bleeding, pregnancy, and childbirth. Menorrhagia is a common presenting symptom, often leading to delayed diagnosis and mismanagement [10]. Studies have reported HMB as a frequent presentation in women diagnosed with RBDs [17]. In our study, menorrhagia was identified as the most frequent symptom, occurring in over 74% of cases, which aligns with the findings of Saes et al. [11]. In contrast, Sharma et al. [12] reported mucocutaneous bleeding as the predominant presentation. The high prevalence of heavy menstrual bleeding (HMB) in women with RBDs underscores the need for heightened awareness and early diagnostic intervention [13].

In our study, intracranial bleeding occurred in three patients with FXIII and FV deficiencies, two patients with FVII deficiency, and one patient with FX deficiency. Conversely, an Indian study identified FXIII deficiency as the most frequent cause of intracranial bleeding [18,19].

In our population, a moderate correlation between FV levels and FVII with the ISTH BAT score was found, while there was a weak correlation between FX and FXIII with the ISTH BAT score. There was no correlation between FI, FXI, FXII, and combined FV+FVIII deficiency with ISTH BAT score, while a study conducted by Saes et al. [11] showed a strong correlation between baseline coagulation factor activity level and the ISTH BAT score for FII deficiency and FX deficiency. A moderate correlation was found in fibrinogen, FV, FVII, and FXIII deficiencies. There was no correlation found in FXI deficiency.

Pregnancy in women with RBDs is fraught with potential complications, including miscarriage, antepartum hemorrhage, and PPH. The risk of PPH is particularly high, necessitating meticulous perinatal management and preparedness for aggressive hemostatic interventions [20,21]. Our findings indicate that 8% of pregnancies in FX and 22% in FXIII deficiency were complicated by significant bleeding events. PPH, a major complication in this population, was effectively managed through a multidisciplinary approach that integrated obstetric care with prompt factor replacement therapy.

The foundation of managing RBDs in females lies in individualized hemostatic therapy, with the replacement of specific clotting factors being the mainstay of treatment [22]. Depending on the deficiency, options such as fibrinogen concentrates, prothrombin complex concentrates, and recombinant FVIIa (rFVIIa) are utilized. rFVIIa has demonstrated effectiveness in controlling bleeding episodes and supporting surgical interventions in women with FVII [19]. Early administration of appropriate factor replacement was shown to significantly reduce bleeding complications, emphasizing the critical role of timely intervention [16]. However, access to factor concentrates is limited in resource-poor settings, and their high cost poses a major challenge [23]. As a result, clinicians frequently resort to fresh-frozen plasma (FFP) or cryoprecipitate as alternative options.

Antifibrinolytic agents such as tranexamic acid and epsilon-aminocaproic acid are invaluable adjuncts in managing mucocutaneous bleeding and menorrhagia [17]. These agents stabilize clots and reduce bleeding in non-surgical contexts. In our study, 99% of patients reported satisfactory control of menorrhagia with Antifibrinolytic therapy, demonstrating its efficacy as a first-line treatment for menstrual bleeding.

Hormonal therapies, including oral contraceptives, levonorgestrel-releasing intrauterine devices, and gonadotropin-releasing hormone agonists, play a pivotal role in managing menorrhagia. These therapies not only reduce menstrual blood loss but also provide contraception, which is crucial for preventing high-risk pregnancies [24]. Our results showed that 40% of women with RBDs benefited from hormonal management, with a significant reduction in the frequency and severity of menorrhagia. Hormonal therapy, used alongside hemostatic treatment, proved beneficial for women with menorrhagia, significantly reducing menstrual blood loss and enhancing the quality of life for most patients.

The complexity of RBDs necessitates a multidisciplinary approach involving hematologists, gynecologists, obstetricians, and specialized nursing staff [25]. Comprehensive care plans tailored to individual patient needs, particularly around surgical procedures, childbirth, and HMB, are essential [22]. Our study advocates for integrated care pathways that facilitate coordination among specialists, ensuring optimal outcomes for women with RBDs.

## Conclusions

Women with RBDs face a spectrum of bleeding challenges, significantly impacting their quality of life and reproductive health. Early diagnosis and personalized treatment strategies are paramount in mitigating bleeding risks and enhancing patient outcomes. Addressing the clinical and therapeutic complexities of RBDs in females requires heightened awareness, timely diagnosis, and equitable access to care. Continued research and clinical collaboration are essential to refine management protocols and improve the standard of care for this vulnerable population. This study highlights the distinct challenges encountered by females with RBDs, especially in resource-constrained settings. Customized strategies that prioritize diagnostic accuracy, effective treatment, and psychosocial support are essential to enhancing outcomes and overall quality of life. Continued research into gender-specific manifestations and innovative treatment options will be vital in closing current gaps and improving care for this underserved population.
